# Challenging the Vestibular System Affects Gait Speed and Cognitive Workload in Chronic Mild Traumatic Brain Injury and Healthy Adults

**DOI:** 10.3389/fneur.2022.819169

**Published:** 2022-06-23

**Authors:** Linda D'Silva, Prabhakar Chalise, Michael Rippee, Hannes Devos

**Affiliations:** ^1^Department of Physical Therapy, Rehabilitation Science, and Athletic Training, University of Kansas Medical Center, Kansas City, MO, United States; ^2^Department of Biostatistics, University of Kansas Medical Center, Kansas City, MO, United States; ^3^Department of Neurology, University of Kansas Medical Center, Kansas City, MO, United States

**Keywords:** mild traumatic brain injury, persistent symptoms, dynamic visual acuity, cognitive workload, gait speed, usual walk, walking with head turns

## Abstract

People with persistent symptoms after mild traumatic brain injury (mTBI) report imbalance during walking with head movements. The purpose of this study was (1) to compare usual walk gait speed to walking with head turns (HT) between people with mTBI and controls, (2) to compare the cognitive workload from usual walk to HT walk between groups, and (3) to examine if gaze stability deficits and mTBI symptoms influence gait speed. Twenty-three individuals (mean age 55.7 ± 9.3 years) with persistent symptoms after mTBI (between 3 months to 2 years post-injury) were compared with 23 age and sex-matched controls. Participants walked a 12-inch wide, 60-foot walkway when looking ahead and when walking with HT to identify letters and their colors. Gait speed during usual walk and HT walk were calculated. Pupillary responses during both walks were converted to the Index of Cognitive Activity (ICA) as a measure of cognitive workload. Gaze stability was examined by the dynamic visual acuity (DVA) test in the yaw plane. The post-concussion symptom scale (PCSS) was used to collect symptom severity. Within group analysis showed that gait speed was lower during HT walk compared to usual walk in the people with mTBI (*p* < 0.001) as well as in controls (*p* < 0.001). ICA was higher with HT compared to usual walk in the mTBI group in the right eye (*p* = 0.01) and left eye (*p* = 0.001), and in controls in the right eye (*p* = 0.01) and left eye (*p* = 0.01). Participants in the mTBI group had slower usual (*p* < 0.001), and HT gait speed (*p* < 0.001) compared to controls. No differences were noted in ICA in the right or left eye during usual walk and HT walk between groups (*p* > 0.05). DVA loss in the yaw plane to the right and left was not different between groups (*p* > 0.05) and were not correlated with gait speed. PCSS scores were correlated with usual walk (*r* = −0.50, *p* < 0.001) and HT gait speed (*r* = −0.44, *p* = 0.002). Slower gait speed, poorer stability, and higher cognitive workload during walking with head turns may reduce community participation in people with mTBI and persistent symptoms.

## Introduction

Mild traumatic brain injury (mTBI) is defined as a “complex pathophysiological process affecting the brain, induced by traumatic biomechanical forces” typically operationalized by a Glasgow Coma Scale of 13–15 (1, 2). Symptoms after a mTBI include dizziness, blurry vision, and imbalance, often due to injury to the vestibular system and its extensive connections with the visual system (3, 4). The number of people with persistent symptoms, including symptoms that evolve or emerge beyond the 3-month period since injury has been reported to be from 15% and up to 82% in the mTBI literature (5–8).

Of the various symptoms noted after chronic mTBI, head turning during walking is shown to have a destabilizing effect on dynamic balance (9). Individuals with vestibular dysfunction have significantly worse postural control, which is evident in dual task conditions where balance and cognitive tasks are combined (10–12). Gait speed and balance control are reported to be poorer in people with mTBI during dual task activities involving balance and cognitive tasks (13–16). A recent study by Gagne et al. had young adults with mTBI who were between 4 and 15 weeks post-injury participate in various locomotor tasks such as level walking, stepping over obstacles, and tandem walking with various cognitive conditions. They report slower gait speed in the mTBI group under dual task conditions (15). However, no studies have included a task such as head turns, which challenges the vestibular system, in combination with a cognitive task such as identifying letters while walking. A dual task of this nature is frequently encountered in daily life while grocery shopping or crossing the street. A lab-based test that mimics activities of daily life may allow us to explore the impact of head turns and consequent influence on balance control.

The vestibular system with calibration from the visual system, is also responsible for maintaining a stable gaze when the head or surrounding environment are moving (17). In people with persistent symptoms after mTBI, reports of blurred vision while driving have been reported by 30% of people (18). Wright et al. examined 14 young adults in the post-acute stage of concussion (within 6 months) and report that visual motion resulted in significantly poorer dynamic balance control compared to controls (19). In young adults with a previous history of concussion (>2 years), greater loss of visual acuity with head movements have been noted as compared to heathy controls (20, 21). However, the effect of gaze instability on balance control during walking has not been explored. The impact of persistent symptoms, gaze instability, and the destabilizing effect of head turns on dynamic balance can increase the mental effort needed to complete daily walking activities.

Cognitive workload is defined as the mental effort that is needed to execute a task (22). When task demand is lower than the cognitive resources, the task is executed accurately. When task performance requires increased cognitive processing, performance is shown to decline (22). Pupillary response has shown to be a reliable and valid measure of cognitive workload in healthy individuals as well as in people with neurological conditions and is responsive to change from single task to dual task postural balance conditions (23–26). Three studies have assessed pupillary changes following brain injury during performance of a cognitive task (27–29). Koelewijn et al. found no changes in task-evoked pupillary response (TEPR) in a speech perception task between individuals with brain injury and controls. However, higher accuracy in the performance of the speech perception task was associated with greater pupil dilation (28). Ayala and Heath revealed larger TEPR during anti-saccade movements in patients with a history of concussion compared to controls (27). Tapper et al. extended the findings of the previous studies by comparing mean pupillary diameter during dual-tasking between individuals without and with concussion. They found that individuals with a history of concussion exerted larger mean pupillary size during tasks of lower cognitive demand, compared to controls (29). Although there is encouraging evidence that pupil dilation can be used as a sensitive measure of cognitive workload in mTBI, no studies have evaluated pupillary responses in dual task walking conditions.

Therefore, the purpose of this study was (1) to compare the gait speed during usual walk and walking with head turns (HT) while performing a cognitive task between people with mTBI and controls; (2) to examine the associated cognitive workload measured by pupillary response during the usual walk and walk with HT, and (3) to examine the relationship between vestibular function (measured by gaze stability), symptom severity [measured by the post-concussion symptom scale (PCSS)], and gait speed. Our hypotheses were that because of symptoms experienced and gaze instability (1) people with mTBI will have decreased gait speed during usual walk which will further decrease during walk with HT and the cognitive task compared to controls, (2) people with mTBI will show increased cognitive workload, indexed by pupillary response, during usual walk which will further increase during walk with HT and cognitive task compared to controls, and (3) PCSS scores and gaze instability will correlate with usual and HT gait speed.

## Materials and Methods

### Study Design

This was a cross-sectional, comparative study conducted at the University of Kansas Medical Center. The study protocol was approved by the University's Institutional Review Board.

### Participants

Most participants with mTBI were recruited from the Neurology clinic, with the assistance of a neurologist (MR) (*n* = 21). Additionally, the Healthcare Enterprise Repository for Ontological Narration (HERON) (30, 31) search discovery tool was used to identify persons with mTBI who were seen at the university hospital and who met inclusion and exclusion criteria (*n* = 2). Participants were included if they were: (1) Between 40 and 80 years of age, (2) Had a diagnosis of mTBI coded by ICD-10 (S06.0X0A- S06.0X9S) criteria, which include a history of traumatic brain injury and the presence of 3 or more of the following 8 symptoms: (1) headache, (2) dizziness, (3) fatigue, (4) irritability, (5) insomnia, (6) concentration or (7) memory difficulty, and (8) intolerance of stress, emotion, or alcohol. (3) Had persistent symptoms from their injury (determined with the PCSS, a subjective self-report), (4) Were between 3 months to 2 years since their injury. The time since injury was determined with feedback from the neurologist based on patient population seen in the clinic.

Participants with mTBI were excluded if they (1) Had a diagnosed neurological problem such as stroke, Parkinson's disease, Multiple Sclerosis; (2) History of a visual disorder prior to the injury such as cataracts; (3) History of vestibular disorder such as vestibular neuritis, Meniere's disease prior to the mTBI, (4) Had lower extremity injury, recent surgery or pain that would impact the walking tests, (5) Had a history of cancer and received chemotherapy, or (6) If they were involved in litigation due to the injury. Exclusion criteria 5 was based on the independent effect of chemotherapy on the vestibular system (32, 33), and 6 was based on increased stress levels in people involved in litigation which may affect performance (8).

Healthy controls with no history of mTBI were recruited through word-of-mouth from the campus, and from the community, and were individually matched for sex and age (±5 years). Like participants with mTBI, healthy controls were excluded if they had prior neurological disease, visual dysfunction, or pre-existing vestibular disease such as vestibular neuritis or Meniere's disease; if they had lower extremity pain or recent surgery; and had a history of cancer and received chemotherapy.

### Study Procedure

Participant eligibility was verified using a phone screen and eligible participants were scheduled for a testing session. All participants were informed to wear comfortable shoes and bring their corrective eyewear to the testing session. After completing informed consent, demographic information, medical history such as height, and weight; manual muscle test and sensory testing were completed. For people with mTBI, the date of injury was collected.

#### Walking Tests

**Figure 1:**
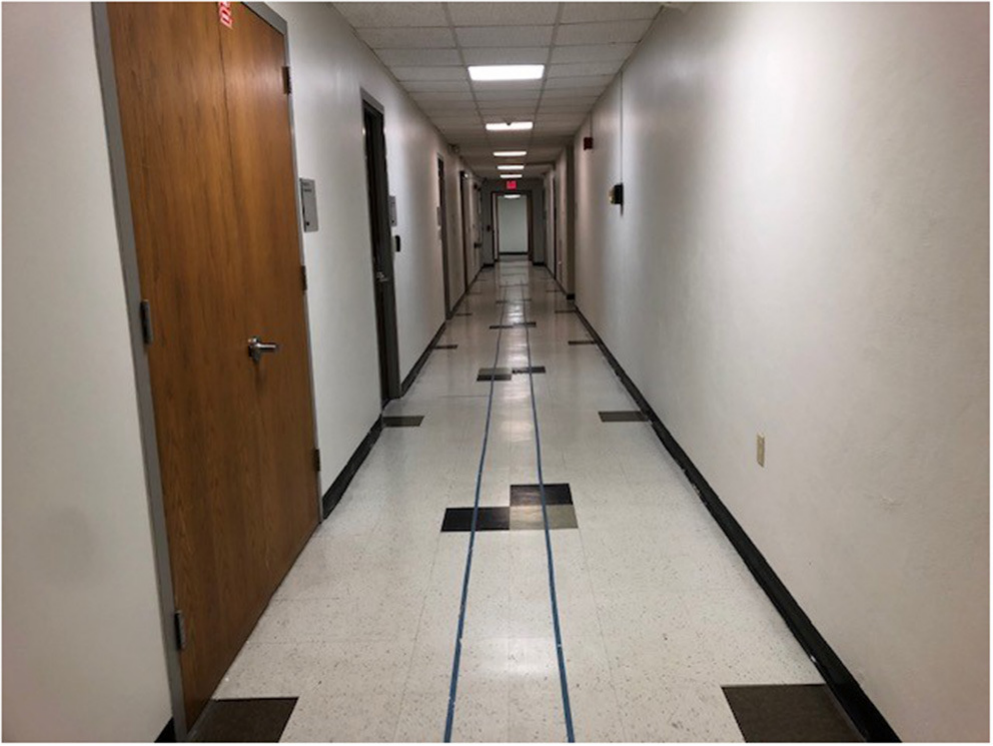
Experimental setup of the walking path which was 60 feet long and 12-inches wide. Photograph used with permission.

The walking tests were conducted in a quiet hallway with no windows and consistent lighting where participants had to walk a 60-foot walkway that was 12-inches wide and marked by tape (Figure 1). Before initiating the tests, participants were informed of the two walking conditions and asked to identify letters and colors to assure that they did not have color blindness. First, they performed 3 trials while looking ahead with instructions to stay within the 12-inch path to the best of their ability. Next, participants performed 3 trials of walking with head turns from side to side to identify letters that were 1.5 inches in size and their colors. In this motor-cognitive dual task activity, there were 12 letters that were affixed ~5 feet apart from each other on the walls of the hallway. Participants were instructed to turn their head to identify the letters and colors instead of reading the letters from a distance. The first trial started at one end of the walkway while the second trial started from the other end, hence they could not memorize the letters by the third trial. Time to walk the path, steps outside the path, and number of missed letters were collected for each trial and the average is reported. The entire foot had to be outside of the taped path to be considered “outside the path.” Gait speed was calculated for usual walk and HT walk as (18.28 meters/time to walk the path) in meters/second.

#### Index of Cognitive Activity (ICA)

Participants wore the Tobii Pro 2 glasses (Tobii Technology AB Sweden.) to capture pupillary responses during the walking tests. Before each walking test, the glasses were calibrated with the participant focusing on the center of the calibration target which was affixed to the wall at eye level. Participants stood between 3 and 4 feet from the wall during the calibration and had to focus on the target until the calibration process was completed. After completing the walking tests, the pupillary response was extracted at 60 Hz using EyeWorks Analyze™ (Eye Tracking LLC, California, USA) software to calculate the Index of Cognitive Activity (ICA). Conventional measures of pupillary response that compare the averaged raw pupillary diameter after stimulus onset to the averaged baseline pupillary diameter (i.e., TEPR), pose some challenges. First, the light reflex may confound extraction of the TEPR, especially in experimental conditions where ambient lighting or luminosity of the screen settings cannot be entirely controlled (34). Second, changes in camera angle and eye movements may interfere with raw pupillary recording (35, 36). The Index of Cognitive Activity (ICA) is calculated from the number of rapid changes in pupillary diameter rather than the difference between averaged pupillary diameter before and after stimulus onset (37, 38). The ICA computes the average number of abrupt discontinuities in pupil size per second and transforms these values into a continuous scale ranging between 0 (no cognitive workload) and 1 (maximum workload). The average ICA of the 3 walking trials for each walking condition has been reported.

#### Dynamic Visual Acuity

The Bertec^®^ Vision Advantage™ (Bertec^®^ Corporation, Columbus, Ohio, USA) was used to administer the Dynamic Visual Acuity Test (DVAT). It includes a wireless inertial measurement unit mounted in the center of the participant's forehead using an elastic headband with a 3-axis integrating gyro (Yost 3-Space Wireless Sensor, Yost Labs, Portsmouth, Ohio, USA) to determine rotational head velocity in the yaw and pitch planes (39). Details of testing have been described in a previous paper (9). In brief, visual acuity was determined in a static head position followed by perception time testing. Based on these parameters, dynamic visual acuity testing was individualized for each participant where they had to generate active rotational head movements to 20 degrees from midline in each direction at a target velocity of 100 degrees per second (with a range from 85 to 120 degrees/s). The outcome variable for the DVAT was loss of lines in logMAR, calculated as the difference between dynamic and static visual acuity, to the right and left in the yaw plane. Higher logMAR values indicate poorer dynamic visual acuity, with loss of more than 0.2 logMAR (>2 lines of loss) considered as clinically significant (40, 41).

#### Symptom Severity

The post-concussion symptom scale (PCSS) is a 22-item self-report measure of symptoms experienced. The severity of symptoms experienced is rated on a Likert scale from 0-indicating “no” symptom to 6-indicating “severe” complaint. The maximum PCSS score is 132 with higher scores reflecting either more symptoms or higher severity of symptoms (42, 43). The PCSS has 4 subgroups; somatic, emotional, cognitive, and sleep.

### Statistical Analysis

Data were inspected for normality using histograms and the Kolmogorov-Smirnov test of normality. Independent sample *t*-tests were used to compare variables that were normally distributed between groups (age, BMI, DVA loss right, and left in LogMAR, gait speed), while data that was not normally distributed were compared using Mann-Whitney U test (ICA for each eye during usual walk, HT walk, and PCSS). Differences in ICA were assessed between the mTBI and control groups adjusting for gait speed using multiple linear regression analysis. Log transformation was used on ICA to satisfy the normality assumption. The analyses were carried out for both usual walk and HT walk and for the right and left eye separately. Paired samples *t*-tests were used to compare usual and HT gait speed within groups while Wilcoxon signed rank tests were used to compare usual and HT walk ICA values within groups. Pearson's correlations were used to examine the relationship between DVA loss and gait speed in both conditions where the data satisfied normality assumptions, while Spearman's rank correlations were used to examine the relationship between PCSS and gait speed in both conditions where data did not satisfy normality assumptions. Correlations were interpreted as fair (0.25–0.50), moderate (0.5–0.75), and good (>0.75) (44). All statistical analyses were conducted using SPSS for Windows version 25.0 (SPSS Inc., Chicago, USA) and *p*-value <0.05 were considered statistically significant.

## Results

### Participant Characteristics

Forty-six individuals completed the study: 23 in the mTBI group (19 females and 4 males) and 23 age and sex-matched controls. The mean duration since injury was 33.2 ± 5.1 weeks (range: 12–92 weeks). There were no differences in demographics between the groups, participants with mTBI had higher PCSS scores (*p* < 0.001) compared to controls. Three control subjects had diagnosed hearing loss (two were genetic) and three had a prior history of migraines. In the mTBI group, two participants complained of tinnitus since the injury, two had a prior history of migraines, and three were wearing prescription glasses with prisms. No strength deficits were noted with manual muscle testing, sensation in the feet was impaired in one control and two persons with mTBI (Table 1).

**Table 1:** Participant characteristics between mTBI and control groups.

	**mTBI group**	**Control group**	***p*** **-value**
	**(*n* = 23)**	**(*n* = 23)**	
Age (years)^a^ (mean ± SD)	55.70 ± 9.3	55.13 ± 9.1	*p* = 0.84
Sex (female/male)	19/4	19/4	
BMI (kg/m^2^)^a^ (mean ± SD)	31.4 ± 7.9	28.77 ± 6.5	*p* = 0.22
Weeks since injury	33.23 ± 5.1	NA	
Right DVA loss (LogMAR)^a^ (mean, SD)	0.21 ± 0.11	0.20 ± 0.09	*p* = 0.78
Left DVA loss (LogMAR)^a^ (mean, SD)	0.21 ± 0.09	0.21 ± 0.11	p = 0.98
Post-concussion Symptom Scale^b^ (median, range)	58.50 (9–110)	2 (0–37)	*p* < 0.001^*^

*mTBI, mild traumatic brain injury; DVA, dynamic visual acuity*.

a *Indicates comparisons using independent sample t-tests*.

b *Indicates comparisons using Mann–Whitney U test*.

* *Indicates significant differences between groups*.

### Single and Dual-Task Gait and ICA Characteristics

**Figure 2:**
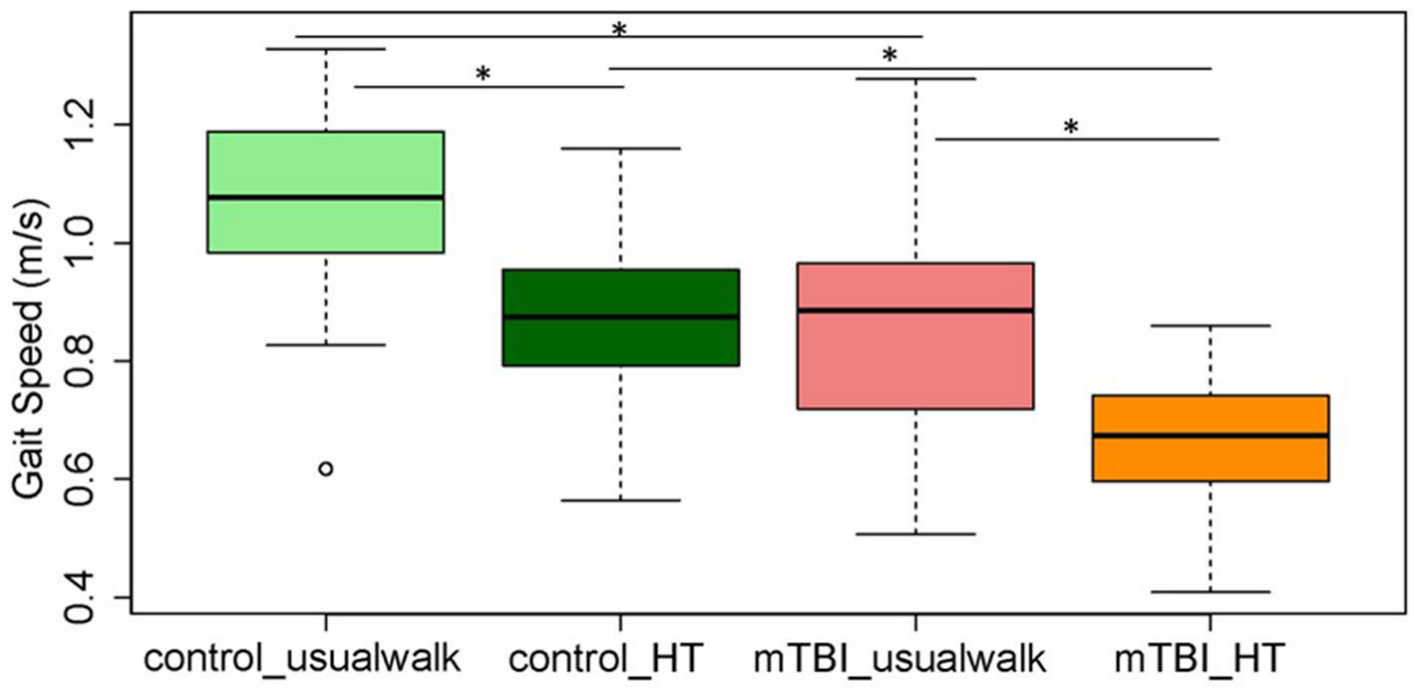
Comparisons of average gait speed between and within the mTBI and control groups during usual walk and walking with head turns. *Indicates significant differences between and within groups. mTBI, mild traumatic brain injury; HT, head turns.

**Figure 3:**
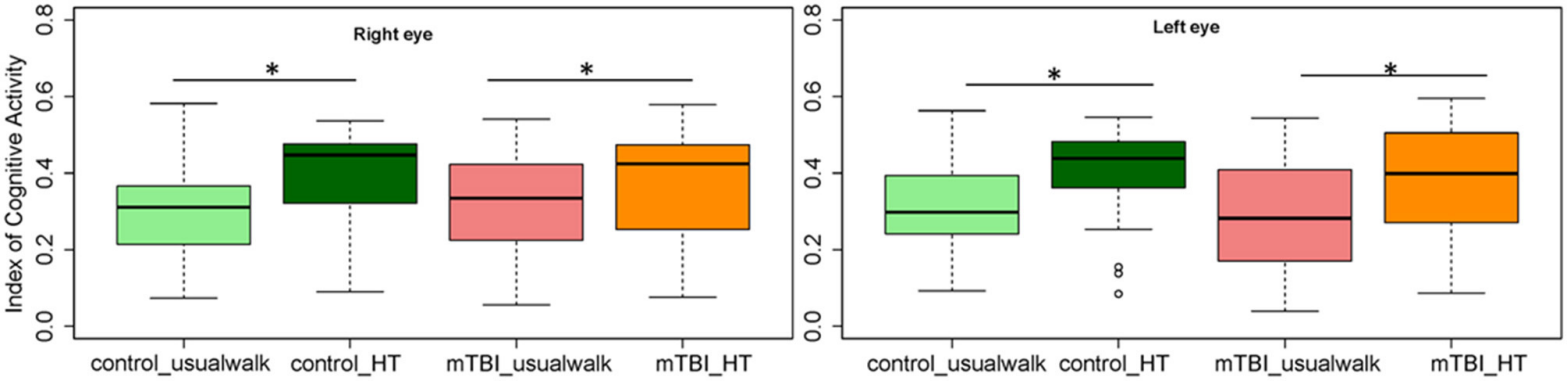
Comparisons of the Index of Cognitive Activity of the right and left eye within groups and between groups in the usual walk and head turn walk conditions. mTBI, mild traumatic brain injury; HT, head turn. *Indicates significant differences within groups.

Within group comparisons show that HT gait speed was lower compared to usual gait speed in the control (*p* < 0.001) and the mTBI group (*p* < 0.001) (Figure 2). The ICA was higher with HT compared to usual walk for controls in the right eye (*p* = 0.01) and left eye (*p* = 0.01) and for people with mTBI in the right eye (*p* = 0.01) and left eye (*p* = 0.001) (Figure 3).

**Figure 4:**
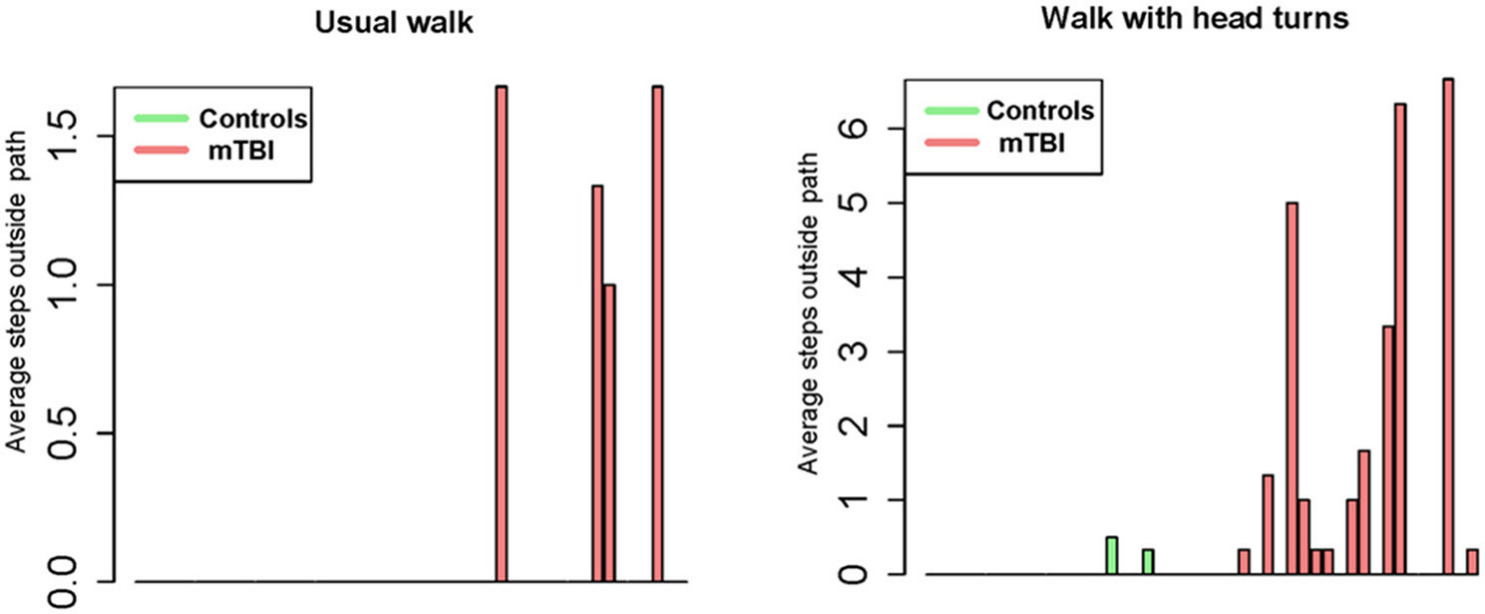
Bar graph representation of steps taken outside the 12-inch pathway during usual walk and walking with head turns between controls and participants with mTBI.

**Figure 5:**
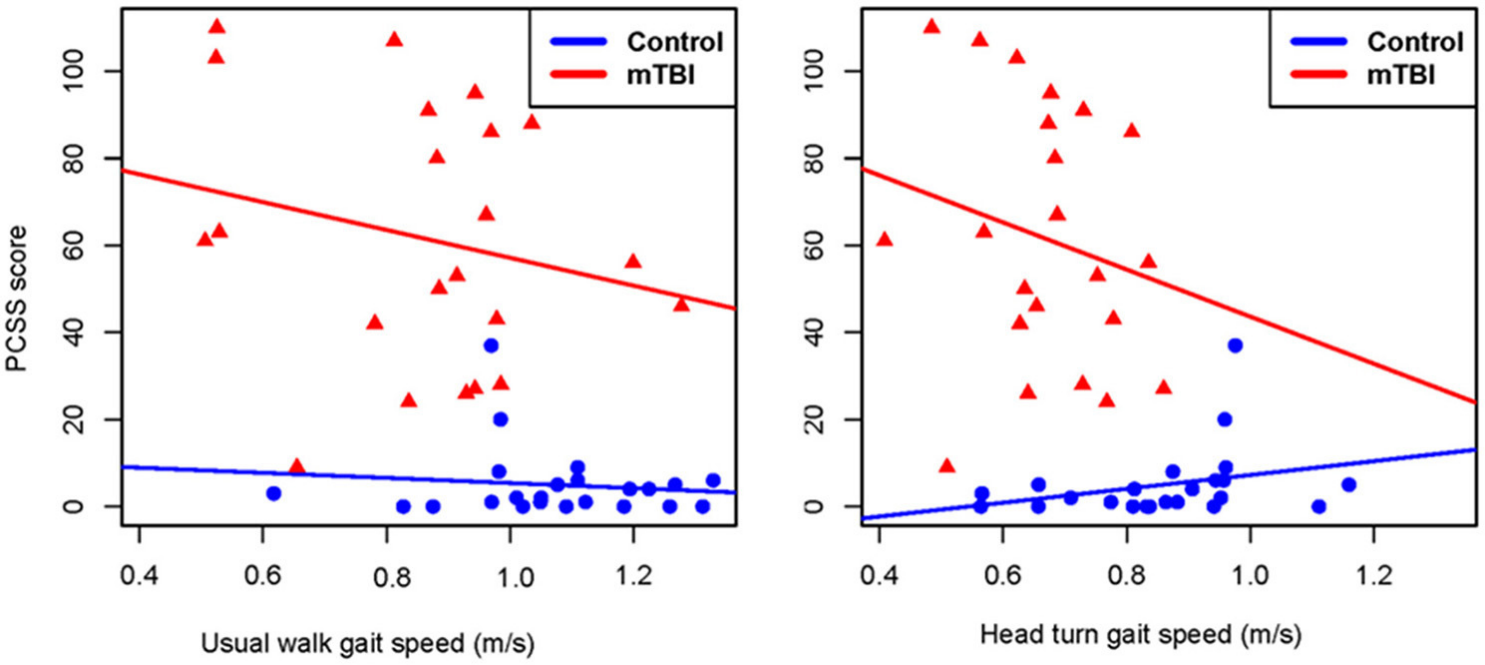
Correlation plots showing the relationship between gait speed during usual walk and head turn walk and post-concussion symptom severity in people with mild traumatic brain injury and controls. PCSS, post-concussion symptom scale; mTBI, mild traumatic brain injury.

Between group comparisons show that participants with mTBI had slower usual gait speed (*p* < 0.001), slower HT gait speed (*p* < 0.001) (Table 2, Figure 2) and took more steps off the path during usual walk and HT walk compared to controls (Figure 4). Participants with mTBI missed more letters (range: 0–5) compared to controls (range: 0–1.3), *p* = 0.48. The ICA for the right and left eye were not different between groups (Figure 3). After adjusting for differences in usual walk gait speed, ICA was not different between the mTBI and control groups for the right eye (*p* = 0.7) or left eye (*p* = 0.51). Likewise, no differences were seen in ICA after adjusting for baseline HT gait speed for the right (*p* = 0.9) or left eye (*p* = 0.7). Dynamic visual acuity in the yaw plane was not different between groups (*p* > 0.05). Correlations between right DVA loss and usual walk gait speed (*r* = 0.16, *p* = 0.29), left DVA loss and usual walk gait speed (*r* = −0.05, *p* = 0.75), right DVA loss and HT gait speed (*r* = 0.26, *p* = 0.08), and left DVA loss and HT gait speed (*r* = 0.22, *p* = 0.14) were not significant. Correlations between right eye ICA and usual walk speed (*r* = −0.08, *p* = 0.59), left eye ICA and usual walk speed (*r* = −0.03, *p* = 0.87), right eye ICA and HT gait speed (*r* = 0.07, *p* = 0.66) and left eye ICA and HT gait speed (*r* = 0.12, *p* = 0.44) were not significant, however, PCSS score was moderately correlated with usual gait speed (*r* = −0.5, *p* = 0.001) and HT gait speed (*r* = −0.44, *p* = 0.002) (Figure 5). All subgroups of the PCSS were correlated with gait speed (*p* < 0.05). The somatic subgroup showed moderate correlations with usual walk (*r* = −0.57, *p* < 0.001) and HT gait speed (*r* = −0.55, *p* < 0.001), and the remaining subgroups showed fair correlations.

**Table 2:** Differences in gait speed and cognitive workload between participants with mTBI and controls.

	**mTBI group (*n* = 23)**	**Control group (*n* = 23)**	***p*** **-value**
Usual walk gait speed (m/s)^a^	0.86 ± 0.21	1.08 ± 0.17	*p* < 0.001^*^
Head turn gait speed (m/s)^a^	0.67 ± 0.11	0.86 ± 0.16	*p* < 0.001^*^
ICA- right eye-usual walk^b^ (median, IQR, 95% CI)	0.33 (0.24) (0.26, 0.38)	0.31 (0.16) (0.25, 0.36)	*p* = 0.59
ICA- right eye-HT walk^b^ (median, IQR, 95% CI)	0.42 (0.22) (0.29, 0.43)	0.45 (0.16) (0.33, 0.45)	*p* = 0.56
ICA- Left eye-usual walk^b^ (median, IQR, 95% CI)	0.28 (0.26) (0.22, 0.35)	0.29 (0.17) (0.26, 0.36)	*p* = 0.68
ICA- Left eye-HT walk^b^ (median, IQR, 95% CI)	0.39 (0.27) (0.31, 0.44)	0.44 (0.14) (0.33, 0.45)	*p* = 0.96

a *Indicates comparisons using independent sample t-tests and is expressed as mean ± standard deviation*.

b *Indicates comparisons between groups based on Mann–Whitney U test and is expressed as median, interquartile range, and 95% CI*.

* *Indicates significant differences between groups. One person in the control group had missing ICA data*.

## Discussion

In this study we examined gait speed in usual walk and walking with head turns while performing a cognitive task and explored the associated cognitive workload in each condition, the effect of gaze instability, and symptom severity on gait speed in people with persistent symptoms after mTBI. Results of this study show that during the performance of a challenging walking task where people had to walk within a specified narrow path, those with mTBI and higher symptom severity had significantly slower gait speed compared to age matched controls. The walking with head turns that included a cognitive task of naming letters and colors resulted in both groups reducing their gait speed, however, the mTBI group continued to have significantly lower gait speed compared to the control group. Pupillary response, reported by the Index of Cognitive Activity (ICA) increased from usual walk to the walk with HT condition in both groups, however, did not differ between groups. Gaze stability did not correlate with gait speed in either walking condition, however higher severity of post-concussion symptoms was associated with slower gait speed in both walking conditions.

Gait speed is an important measure of function and a powerful predictor of quality of life, disability, survival, cognitive decline and falls (45). However, walking as an activity is usually combined with cognitive tasks in daily life such as grocery shopping, walking across the street, or in a park where head turns are necessary. Studies in the younger mTBI population due to sports related injury have shown that motor-cognitive dual tasks result in slower gait speed immediately after injury (46, 47), as well as in the chronic stage of injury (14, 15, 48–50). Gagne et al. found slower gait speed in young adults (average age 22 years) compared to age matched controls, during various walking and cognitive dual tasks, although their subjects had normal cognitive test results and were considered “recovered” with no persistent symptoms (15). Likewise, Fino et al. found that concussed athletes (18–20 years of age, *n* = 4) had larger dual task costs in turning speed and stride time compared to controls when they were examined within 6 weeks of injury (46).

Studies in the middle-age and older adult population are limited but nonetheless very important as these age groups face different challenges after injury. Results of this study show that gait speed (age: 45–65 years) was significantly slower in the mTBI population and it decreased further with head turns and the cognitive task, compared to controls. On average, the gait speed in the mTBI group with HT walking was 0.67 m/s compared to 0.86 m/s during usual walk, which reflects the task difficulty of staying in a narrow path while scanning and walking. Fino et al. examined gait speed and turning dynamics in 14 adults (average age 38 years) with persistent symptoms after mTBI (>3 months post-injury) while walking laps. They report that participants with chronic mTBI had slower gait speed and impaired head stabilization during turning compared with controls which was correlated with higher symptom severity (47). The association between post-concussion symptoms, gait speed, and dynamic balance has been studied. Our group has shown that higher symptom severity is associated with poorer performance on the functional gait assessment, a test of dynamic balance in chronic mTBI (9). Kleffelgaard et al. report higher symptom severity (measured by the Rivermead post-concussion questionnaire) was associated with persistent gait and balance deficits measured by gait speed, the dynamic gait index and the 6-min walk test, 4 years after injury (51), and people with mTBI (3 months post-injury) who experienced more dizziness related disability had poorer performance on balance (Balance Error Scoring System) and mobility (HiMAT) tests (52). Results of this study confirm previous study findings, showing that higher symptom severity is associated with slower gait speed.

Our second objective was to examine the cognitive workload during usual and HT walking conditions. Our hypothesis was that walking with HT and a dual task would require more cognitive workload, indexed by the ICA, compared to the usual walk condition. Study results showed that cognitive workload increased from the usual walk to the HT condition within each group. Our results are similar to Kahya et al. who examined ICA during standing with eyes open and occluded and with dual tasking. They reported increased cognitive workload with eyes occluded and with dual tasking which was correlated with higher postural sway (24). However, our results differ from Tapper et al. who report that with increasing task difficulty, asymptomatic athletes with a sports-related concussion had poorer behavioral responses but did not demonstrate an increase in pupil dilation when compared to the easier single task and to control subjects. They suggest that individuals with concussion reach their cognitive capacity limits earlier and with easier tasks with an inability to recruit more cognitive resources leading to deterioration in task performance as demand increases (29). Likewise, Koelewijn et al. found no differences in pupil dilation with increasing task difficulty in the acute stage of mTBI suggesting that depleted resources due to increased distractibility and higher fatigue levels result in cognitive overload relatively early (28). A reason for the difference between our results and the studies mentioned above is that we examined ICA as a moment-to-moment change in pupillary response whereas Tapper et al. and Koelewjin et al. looked at mean pupillary size and baseline corrected pupillary size, respectively. Vogels et al. found that ICA and baseline corrected pupillary size respond differently to changes in task demand and dual tasking in healthy individuals. They report that although pupil dilation increases with task difficulty and dual tasks, the ICA showed a decrease during dual tasks (53). This collective information suggests that we are comparing different constructs of cognitive workload which may explain the discrepancy.

No differences in pupillary responses were found between mTBI and controls in usual walk or HT walk conditions, even after adjusting for baseline differences in gait speed. We hypothesized that symptom burden and gaze instability in the mTBI group would require more cognitive workload to perform the HT motor-cognitive dual task. We examined gaze stability using the dynamic visual acuity (DVA) test, which is a functional measure of the vestibulo-ocular reflex (VOR). A difference of more than 0.2 LogMAR on the dynamic visual acuity test is indicative of gaze instability, with previous research reporting persistent gaze stability deficits in chronic mTBI (20, 21, 47, 52). However, we did not find differences in DVA loss between the mTBI and control groups, because the control group exhibited gaze stability deficits, resulting in non-significant differences between groups. This may be one reason why cognitive workload was not different between the groups. Symptom burden was significantly higher in the mTBI group and may be reflected in the ICA variability seen in the mTBI group. Ultimately, our study results did not show a difference in ICA between the mTBI and control groups, indicating that either ICA is not sensitive enough to differentiate between mTBI and healthy controls or the task was not complex enough to result in a significant change in ICA values. Future studies that include a moving platform that requires participants to maintain a certain speed along with randomly presented visual tasks may increase task complexity enough to detect larger changes in ICA.

Although cognitive workload was similar between groups, participants with mTBI had slower gait speed, poorer balance indicated by steps off the path, and more missed letters during HT walking compared to controls, indicating poorer performance. Devos et al. have reported that people with multiple sclerosis and impaired cognitive function did not increase their cognitive workload but showed a deterioration in functional performance compared to those without cognitive impairment and healthy controls (54). It is possible that people with mTBI are unable to effectively allocate cognitive resources to compensate for decreased performance in walking tests.

Our third objective was to examine if vestibular function, measured by gaze stability, was associated with gait speed. Vestibulo-ocular dysfunction is common after mTBI (19, 55, 56), therefore we expected to see greater DVA loss in the mTBI group compared to controls. We found DVA loss of >0.2 logMAR in 56% of controls and 65% of mTBI participants. We did not find correlations between DVA loss and gait speed and DVA loss and symptom severity. One reason for these results may be the exclusion criteria. We did not exclude control participants with neck pain and did not assess for neck range of motion. Fino et al. examined turning dynamics in 14 individuals (average age 38 years) with chronic mTBI. They found that participants with mTBI had slower gait speed, and impaired turning dynamics compared to controls. Thirty percent of their mTBI participants had impaired gaze stability measured by the video head impulse test, however, their control group was younger (mean age 25.6 years) and had no vestibular dysfunction (47). Kleffelgaard et al. found that 62% of their mTBI subjects had positive findings during oculomotor tests and 29% had DVA loss, however the relationship between vestibular function and performance measures of balance and mobility were not examined (52). Future studies with stringent inclusion/exclusion criteria are necessary to examine how vestibular dysfunction may affect gait speed, cognitive workload, and eventually recovery with training.

This study has several limitations. The main goal was to use an ecologically valid test that included walking with head turns, however, to encourage participants to turn their head we also included a cognitive task of identifying letters and their colors. We tested participants for color blindness before the walking test, however, we did not assess cognitive skills such as working memory, processing speed or executive function that may be affected after mTBI. Cognitive deficits in these domains are common after mTBI and can impact gait speed and dynamic balance. Likewise, mood profiles such as depression and anxiety can affect gait speed and these data were not collected. The walking tests were not randomized; hence participants may have slowed down during the head turn walking tests due to tiredness. In order to track steps outside the path, we taped the narrow walkway, however, the taped path may have resulted in participants slowing down to stay within the path. We emphasized and demonstrated to each participant that head turns were required when they were close to the letter and to avoid looking at the letters ahead of time with eye movements only. However, some subjects may have not turned their head as much which may have influenced gait speed. In this study, neck range of motion was not captured, hence future studies need to examine the extent to which people with mTBI move and/or restrict head movement, and the effect on gait speed. Several subjects in the study wore glasses and the TOBII glasses used to measure pupil dilation had the capability to be fitted according to the subject's needs, but we were not able to match the prescription accurately since some participants with mTBI wore prisms. We did not assess eye movements such as smooth pursuit, and saccades and did not examine visual function for tropias and phorias which could impact the ability to see clearly. We examined vestibular function using the dynamic visual acuity test, which is a functional measure of gaze instability and is dependent not only on the effort the subject puts forth but also on factors such as neck pain. We did not assess vestibular function physiologically, hence future studies that examine vestibular evoked potentials, the video head impulse test, and videonystagmography to quantify otolith, semicircular, and visual function are necessary. Retrospective sample size analysis showed that we had adequate sample size to detect usual walk (91% power) and HT gait speed (97% power) differences between the mTBI and controls, however, ICA was not adequately powered. Future studies with greater task complexity will allow for a closer analysis of the relationship between the visual-vestibular interaction, symptom presentation, cognitive workload, and gait.

## Conclusion

People with persistent symptoms after a mild traumatic brain injury have slower usual gait speed compared to age-matched controls months after the injury. With head turns and an added cognitive task, their gait speed decreased further and continued to be significantly slower than healthy controls. Gait speed which is a marker of health and disability was associated with higher symptoms experienced. These results have important implications for people with mTBI as they return to work, leisure, and community activities.

## Data Availability Statement

The raw data supporting the conclusions of this article will be made available by the authors, without undue reservation.

## Ethics Statement

The studies involving human participants were reviewed and approved by IRB at the University of Kansas Medical Center. The patients/participants provided their written informed consent to participate in this study.

## Author Contributions

LD'S: conception of study, data collection, data analysis, writing, reviewing, and revising of manuscript. PC and HD: conception of study, data analysis, writing, reviewing, and revising of manuscript. MR: recruitment for study, reviewing, and revising of manuscript. All authors contributed to the article and approved the submitted version.

## Funding

This work was supported by internal grants from the Department of Physical Therapy and Rehabilitation Science and the School of Health Professions at the University of Kansas Medical Center. REDCap at University of Kansas Medical Center was supported by CTSA grant from NCRR and NCATS awarded to the University of Kansas Medical Center for Frontiers: University of Kansas Clinical and Translational Science Institute.

## Conflict of Interest

The authors declare that the research was conducted in the absence of any commercial or financial relationships that could be construed as a potential conflict of interest. The handling editor is currently co-organizing a Research Topic with one of the authors HD, and confirms the absence of any other collaboration.

## Publisher's Note

All claims expressed in this article are solely those of the authors and do not necessarily represent those of their affiliated organizations, or those of the publisher, the editors and the reviewers. Any product that may be evaluated in this article, or claim that may be made by its manufacturer, is not guaranteed or endorsed by the publisher.
